# Supplementary material for: Environmental DNA allows upscaling spatial patterns of biodiversity in freshwater ecosystems
Source: Nat Commun. 2020 Jul 17;11:3585. doi: 10.1038/s41467-020-17337-8 (PMC7367889; doi:10.1038/s41467-020-17337-8)
Supplement: Supplementary file 1 — Supplementary Information [file 41467_2020_17337_MOESM1_ESM.pdf]

Supplementary Information

Environmental DNA allows upscaling spatial patterns of biodiversity in freshwater ecosystems

Carraro et al.

Supplementary Tables

Supplementary Table 1. List of land cover, geological, and morphological covariates used.

Type	Acronym	Covariate	Extent
Land cover	L-FO	Forest	Local
Land cover	L-RO	Rocks	Local
Land cover	L-UR	Urban area	Local
Land cover	L-OR	Orchard	Local
Land cover	L-SW	Swamp	Local
Land cover	L-LA	Lake	Local
Geological	G-AL	Alluvial rocks	Upstream
Geological	G-MO	Moraines	Upstream
Geological	G-AP	Alpine sediments	Upstream
Geological	G-WA	Superficial waters	Upstream
Geological	G-LO	Loess	Upstream
Geological	G-SC	Scree	Upstream
Geological	G-PE	Peat	Upstream
Morphological	M-US	Mean upstream channel slope	Upstream
Morphological	M-DA	Drainage area	Upstream
Morphological	M-LS	Local channel slope	Local
Morphological	M-LE	Local elevation	Local
Morphological	M-SO	Stream order	Local

Supplementary Table 2. List of symbols used.

Symbol	Definition	Dimension
$\mathbf{1}_a(x)$	Indicator function, equal to 1 for each element of x that satisfies the condition a, and null otherwise	-
A_i	Drainage area at node i	L^2
$A_{S,i}$	Source area at node i ($A_{S,i} = L_i w_i$)	L^2
$\hat{C}_i$	Modelled eDNA concentration at node i	NL^{-3}
D_i	Water depth at node i (mean value during sampling days)	L
d_i	Sum of squared residuals of observed read numbers N_{io} at node i with respect to $\tilde{r}_{jo}^{(50)}$	-
$\tilde{d}_{ih}$	Sum of squared residuals of generated triplet of read numbers $\widetilde{N}_{iho}$, $o = 1, 2, 3$ at node i with respect to $\tilde{r}_{jo}^{(50)}$	-
f	Likelihood function	-
$\boldsymbol{I}$	Identity matrix	-
k	(Genus-specific) expected read number corresponding to unit eDNA concentration	L^3N^{-1}
L_i	Length of reach corresponding to node i	L
L_{ij}	Length of the along-stream path joining node i to j	L
$\boldsymbol{N}$	Matrix of observed read numbers (of size number of sampling sites by number of replicates) for a given genus	-
N_{io}	Read number observed at site i, replicate o	-
$\widehat{N}_i$	Expected number of reads predicted by the model	-
$\widetilde{N}_{iho}$	Generated read number from the distribution $\mathrm{Geom}(\widehat{N}_i)$ (realization h, replicate o)	-
$\widehat{N}_{U,i}$	Expected read number in an unconnected reach i	-

Symbol	Definition	Dimension
P_{ij}	Set of nodes constituting the along-stream path joining i to j (including i and j)	-
$P_{D,i}$	Detection probability of a genus at node i	-
p	p-value of the two sample Kolmogorov-Smirnov tests performed between distributions of genus richness assessed by eDITH model, eDNA data and kicknet	
p_i	(Genus-specific) eDNA production rate at node i	$NL^{-2}T^{-1}$
p_0	(Genus-specific) characteristic eDNA production rate	$NL^{-2}T^{-1}$
p_i^{GOF}	(Genus-specific) p-value of goodness-of-fit test performed at node i	-
p'_0	(Genus-specific) scale factor for Eq. (3) ($= kp_0$)	LT^{-1}
Q_i	Water discharge at node i (mean value during sampling days)	L^3T^{-1}
q_i	Directly contributing discharge at node i (mean value during sampling days)	L^3T^{-1}
r_{io}	Residual of observed read number N_{io} with respect to $\widehat{N}_i$	-
$\tilde{r}_{iho}$	Residual of generated read number $\widetilde{N}_{iho}$ with respect to $\widehat{N}_i$	-
$\tilde{r}_{iho}^{(50)}$	Median of $\tilde{r}_{iho}$ across all realizations h	-
S	Set of nodes used in calibration	-
s_j	Standard deviation of the triplet of observed read numbers N_{io}, $o = 1, 2, 3$ with respect to $\widehat{N}_i$	-
$\tilde{s}_{jh}$	Standard deviation of the triplet of generated read numbers $\widetilde{N}_{iho}$, $o = 1, 2, 3$ with respect to $\widehat{N}_i$	-
v_i	Water velocity at node i (mean value during sampling days)	LT^{-1}
v_{ij}	Average water velocity along the stream path that connects node i to j	LT^{-1}

Symbol	Definition	Dimension
w_i	River width at node i	L
$\boldsymbol{X}(i)$	Vector of (z-normalized) environmental covariates evaluated at node i	-
$\boldsymbol{\beta}$	(Genus-specific) vector of parameters expressing the effects of covariates $\boldsymbol{X}(i)$ on eDNA production rate p_i	-
$\gamma(i)$	Set of nodes upstream of node i (including i)	-
τ	(Genus-specific) characteristic decay time of eDNA	T

Supplementary Figures

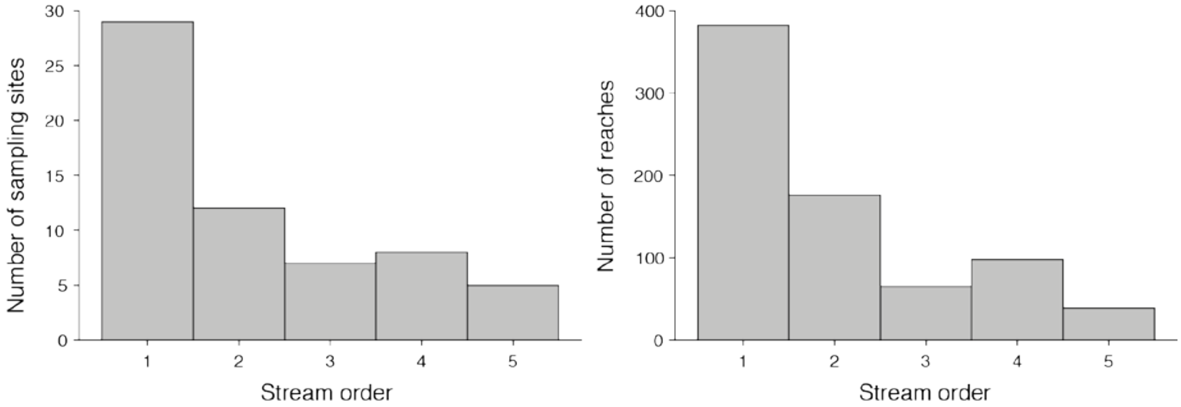

Supplementary Figure 1. Distribution of stream order values across the 61 reaches where eDNA sampling sites were located (left panel) and across the 760 reaches constituting the Thur river network (right panel). The site where eDNA sampling was performed but kicknet was not has stream order value equal to 5.

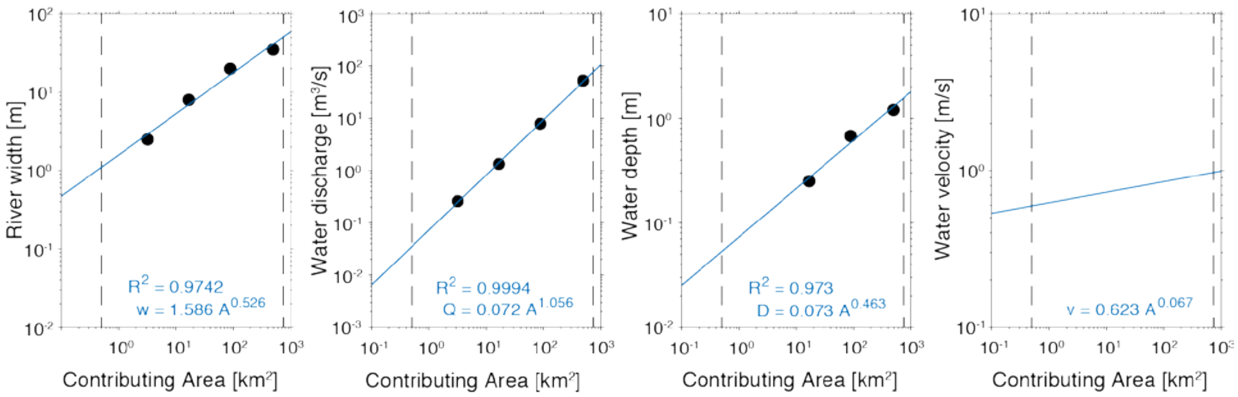

Supplementary Figure 2. Fitting of power-law regressions for the hydrological variables width w (m), discharge Q (m^3s^{-1}), depth D (m) and velocity v (ms^{-1}) against contributing area A (km^2) at the four hydrological stations displayed in Fig. 1a. Dots indicate measured values: measured Q are averages of mean daily values during sampling days (June 11 to June 22, 2016); w are estimated via aerial images, and are assumed to be time-invariant (i.e. river cross-sections are assumed rectangular); D are calculated from stage-discharge relationships starting from the measured Q values. Vertical dashed lines indicate the range of A values across the 760 reaches. The relationship between D and A is fitted on 3 points because the stage-discharge relationship at the hydrological station with lowest contributing area was deemed inaccurate (see Methods). The relationship between v and A is inferred as $v = Q/(Dw)$. Source data are provided as a Source Data file.
