# Supplementary material for: Environmental DNA allows upscaling spatial patterns of biodiversity in freshwater ecosystems
Source: Nat Commun. 2020 Jul 17;11:3585. doi: 10.1038/s41467-020-17337-8 (PMC7367889; doi:10.1038/s41467-020-17337-8)
Supplement: Supplementary file 3 — Reporting Summary [file 41467_2020_17337_MOESM3_ESM.pdf]

nature research

Corresponding author(s): Luca Carraro, Florian Altermatt

Last updated by author(s): Jun 24, 2020

Reporting Summary

Nature Research wishes to improve the reproducibility of the work that we publish. This form provides structure for consistency and transparency in reporting. For further information on Nature Research policies, see Authors & Referees and the Editorial Policy Checklist .

Statistics

For all statistical analyses, confirm that the following items are present in the figure legend, table legend, main text, or Methods section.

n/a	Confirmed	
☐	☒	The exact sample size (*n*) for each experimental group/condition, given as a discrete number and unit of measurement
☐	☒	A statement on whether measurements were taken from distinct samples or whether the same sample was measured repeatedly
☐	☒	The statistical test(s) used AND whether they are one- or two-sided<br>*Only common tests should be described solely by name; describe more complex techniques in the Methods section.*
☐	☒	A description of all covariates tested
☐	☒	A description of any assumptions or corrections, such as tests of normality and adjustment for multiple comparisons
☐	☒	A full description of the statistical parameters including central tendency (e.g. means) or other basic estimates (e.g. regression coefficient) AND variation (e.g. standard deviation) or associated estimates of uncertainty (e.g. confidence intervals)
☒	☐	For null hypothesis testing, the test statistic (e.g. *F*, *t*, *r*) with confidence intervals, effect sizes, degrees of freedom and *P* value noted<br>*Give P values as exact values whenever suitable.*
☐	☒	For Bayesian analysis, information on the choice of priors and Markov chain Monte Carlo settings
☒	☐	For hierarchical and complex designs, identification of the appropriate level for tests and full reporting of outcomes
☒	☐	Estimates of effect sizes (e.g. Cohen's *d*, Pearson's *r*), indicating how they were calculated

Our web collection on statistics for biologists contains articles on many of the points above.

Software and code

Policy information about availability of computer code

Data collection	The data collection was performed as reported in Mächler et al., 2019, doi:10.1002/edn3.33.
Data analysis	Data analyses were performed via MATLAB (version R2018a) custom scripts. The GIS software ArcMap 10.5.1 was used to extract the river network. eDNA sequencing was performed via the following software: FastQC (version 0.11.7), usearch (version 10.0.240), Flash (version 1.2.11), cutadapt (version 1.12), prinseq-lite (version 0.20.4), UNOISE3, R (version 3.5.2), and R-packages taxize (version 0.9.7) and rentrez (version 1.2.2). MATLAB scripts reproducing the results of this manuscript are available at https://doi.org/10.5281/zenodo.3903330.

For manuscripts utilizing custom algorithms or software that are central to the research but not yet described in published literature, software must be made available to editors/reviewers. We strongly encourage code deposition in a community repository (e.g. GitHub). See the Nature Research guidelines for submitting code & software for further information.

Data

Policy information about availability of data

All manuscripts must include a data availability statement. This statement should provide the following information, where applicable:

- Accession codes, unique identifiers, or web links for publicly available datasets
- A list of figures that have associated raw data
- A description of any restrictions on data availability

Sequence data that support the findings of this study have been deposited in European Nucleotide Archive with the study accession numbers (secondary accession number) PRJEB31920 (ERP114535) and PRJEB33506 (ERP116301). Hydrological and landscape data that support the findings of this study are available in Zenodo with the identifier doi:10.5281/zenodo.3903330. Source data are provided with this paper.

Field-specific reporting

Please select the one below that is the best fit for your research. If you are not sure, read the appropriate sections before making your selection.

☐ Life sciences ☐ Behavioural & social sciences ☒ Ecological, evolutionary & environmental sciences

For a reference copy of the document with all sections, see nature.com/documents/nr-reporting-summary-flat.pdf

Ecological, evolutionary & environmental sciences study design

All studies must disclose on these points even when the disclosure is negative.

Study description	In this study, we estimate relative abundance of 50 genera of aquatic insects belonging to the orders Ephemeroptera, Plecoptera and Trichoptera (EPT) at an average resolution of 1-km long river streches by combining eDNA data (collected at 61 locations across the 740-km2-wide study catchment) with a hydrology-based model that accounts for dynamics of local eDNA production, hydrological transport and decay. The model is separately run on data from each of the 50 different genera. These results are then merged in order to provide a prediction of the spatial patterns of biodiversity of aquatic insects at a catchment scale.
Research sample	The dataset used for this study is the one presented in Mächler et al., 2019, doi:10.1002/edn3.33. The choice of focusing on EPT taxa was driven by their well-known high abundance and diversity across the Thur catchment (as confirmed by historical data, see Mächler et al., 2019), as well as for the easiness of collecting individual organisms (existence of standard sampling techniques such as kicknet and availability of taxonomic experience within the study team). The Thur catchment was chosen as case study due to its rather large elevational range (which implies the existence of several habitats with different environmental and hydrological characteristics) and its representativeness for mountainous catchments.
Sampling strategy	The selection of 61 sampling sites was deemed a large enough sample size for a 740-km2-wide basin. The location of sampling sites was heuristically determined in a bid to cover the largest possible range of habitats and hydrological conditions (e.g. stream order values), while respecting the requirement of sites' accessibility.
Data collection	Data were collected by Elvira Mächler and Remo Wüthrich, with the help of Florian Altermatt, Roman Alther, Simon Flückiger, Emanuel A. Fronhofer, Isabelle Gounand, Sereina Gut, Eric Harvey, Samuel Hürlemann and Chelsea J. Little. Kicknets were used to collect individuals at the sampling locations. At the same sites, three indipendent samples of 250 mL of river water were collected and used for subsequent eDNA analyses.
Timing and spatial scale	All eDNA and kicknet data were collected in the period from June 11 to June 22, 2016. Thereby, the data and the subsequent analyses represent a snapshot of the seasonal biodiversity patterns across the catchment. Data were taken at 61 locations spanning a 740-km2-wide catchment.
Data exclusions	No data were excluded from the analyses.
Reproducibility	The results presented in this manuscript are obtained from the analysis of the outputs of a mathematical model, hence they are fully reproducible (see the code at https://github.com/lucarraro/eDITH-thur). Note that, in our manuscript, additional analyses are presented where only a subset of sampling sites is used to inform the model, and the robustness of model outputs to reduction in the extent of data was assessed.
Randomization	Covariates were chosen as representative of all possible environmental drivers potentially controlling the distribution of genera, while satisfying multicollinearity checks: in particular, all pairwise correlation coefficients between covariates were lower than 0.8, while all variance inflation factors were lower than 10. Random allocations were performed in the additional analyses aimed at assessing the effect of reduction in the number of sampling sites in model predictions. In this case, a quasi-random subsampling was performed, where the proportions of sampling sites per stream order value in the subsample was imposed to be equal to that of the complete set of sites. For each of the 3 subsample sizes that were assumed, 3 different subsamples were drawn.
Blinding	In the data analyses, all labels identifying genera and covariates were replaced by numerical identifiers.

Did the study involve field work? ☒ Yes ☐ No

Field work, collection and transport

Field conditions	Sampling was performed during low flow conditions. Temperatures ranged between 15 and 25 Celsius degrees during the days of sampling, and sampling took only place on days without rain (i.e., sampling was halted for at least 24 h after the last rain event).
Location	Sites' location is displayed in Figure 1 of the manuscript. River widths range between about 1 and 50 m; depths of rivers ranged between about 0.1 and 2 m, while sampling was always conducted from the shoreline, and surface water was collected. The coordinates of the river outlet are 47.497085 N, 9.232307 E.
Access and import/export	No permits were required for the kicknet sampling. Access to all sites was legally permitted, as well as collection of organisms. Aquatic invertebrates were collected and stored in 80 % Ethanol. The samples are currently still in the lab of Florian Altermatt for further analyses, but will be permanently stored in the Swiss Data collection for invertebrate samples (CSCF, Neuchâtel). All samples (both invertebrates and eDNA) were only transported and analysed within Switzerland, i.e., their county of origin. Export

	of eDNA samples was performed as detailed in Mächler et al., 2019.
Disturbance	No disturbance to the environment was produced by the field work.

Reporting for specific materials, systems and methods

We require information from authors about some types of materials, experimental systems and methods used in many studies. Here, indicate whether each material, system or method listed is relevant to your study. If you are not sure if a list item applies to your research, read the appropriate section before selecting a response.

Materials & experimental systems

n/a	Involved in the study	
☒	☐	Antibodies
☒	☐	Eukaryotic cell lines
☒	☐	Palaeontology
☒	☐	Animals and other organisms
☒	☐	Human research participants
☒	☐	Clinical data

Methods

n/a	Involved in the study	
☒	☐	ChIP-seq
☒	☐	Flow cytometry
☒	☐	MRI-based neuroimaging

October 2018
