# Supplementary material for: Environmental DNA allows upscaling spatial patterns of biodiversity in freshwater ecosystems
Source: Nat Commun. 2020 Jul 17;11:3585. doi: 10.1038/s41467-020-17337-8 (PMC7367889; doi:10.1038/s41467-020-17337-8)
Supplement: Supplementary file 4 — Description of Additional Supplementary Files [file 41467_2020_17337_MOESM4_ESM.pdf]

Description of Additional Supplementary Files

File Name: Supplementary Data 1

Description: List of EPT genera found in eDNA and kicknet samples and respective ID used in the figures of the main text. Genera are sorted by their accuracy value (see Fig. 7a). ID > 50 indicates genera found in kicknet but not in eDNA. Note that this table presents the same dataset shown in Table S4 of Mächler et al. (2019) (doi:10.1002/edn3.33). However, the latter used a more stringent criterion for local detection of EPT species, only considering genera found in at least 2 out of 3 eDNA replicates. Here instead, it is assumed that 1 out of 3 positive replicates is sufficient to deem the genus as present, as this also considers transport-decay dynamics that are included in eDITH. This leads to slightly higher numbers of detections for the genera *Ameletus*, *Chaetopteryx*, *Ephemerella*, *Ecnomus*, *Ernodes*, *Lepidostoma*, *Pseudopsilopteryx*, and *Rhabdiopteryx*.
